# Developmental profiles of infants with hypoxic ischaemic encephalopathy at a tertiary hospital in South Africa

**DOI:** 10.4102/ajod.v15i0.1729

**Published:** 2026-02-23

**Authors:** Ayanda Myaka-Gama, Sibongile Mbatha, Sarah Lowick, Kebashni Thandrayen, Firdose L. Nakwa

**Affiliations:** 1 Department of Paediatrics and Child Health, School of Clinical Medicine, Faculty of Health Sciences, Chris Hani Baragwanath Academic Hospital, University of the Witwatersrand, Johannesburg, South Africa

**Keywords:** neonates, asphyxia, therapeutic hypothermia, Griffiths Mental Development Scales, general quotient, subscales, neurodevelopmental impairment

## Abstract

**Background:**

Hypoxic ischaemic encephalopathy (HIE) is a common cause of neonatal death and severe neurological deficit in children, contributing to medico-legal litigation.

**Objectives:**

To describe the neurodevelopmental outcomes of infants with moderate and severe HIE at Chris Hani Baragwanath Academic Hospital and the proportions with neurodevelopmental impairment (NDI) and complications. To explore the effect of HIE severity and therapeutic hypothermia (TH) on neurodevelopmental outcome.

**Method:**

A retrospective, descriptive study at the Neonatal Neurodevelopmental Clinic included 239 infants with moderate and severe HIE, between 2015 and 2020. Neurodevelopmental outcomes were assessed by using the Griffiths Mental Developmental Scales at 1 year. General Quotient (GQ) scores defined NDI. Clinical and investigation criteria determined those with neurological complications.

**Results:**

Of the 239 infants, 211 (88.3%) and 28 (11.7%) had moderate HIE and severe HIE, respectively. Cerebral palsy (CP) was diagnosed in 9.2% and NDI in 17.1%. Severe HIE infants had significantly higher rates of NDI and CP, 50% (14) and 21.4% (6) respectively, as compared to those of moderate HIE infants, who had 12.7% (27) NDI and 7.6% (16) CP; 152(72%) moderate and 14 (50%) severe HIE infants received TH. Those who received TH were less likely to have NDI (*p* = 0.005), CP (*p* = 0.002), epilepsy and visual impairment.

**Conclusion:**

Developmental scores at 1 year of age were in the average range for the cohort, with equivalent profiles across domains. Those with severe HIE had the worst outcomes. Therapeutic hypothermia was associated with decreased CP and NDI in both groups.

**Contribution:**

This report supports the use of TH as a neuroprotective strategy in stage 2 and 3 HIE, highlighting the need for neurodevelopmental assessments at 2 years and beyond to determine longer-term outcomes and subtle deficits.

## Introduction

Hypoxic ischaemic encephalopathy (HIE) is a heterogeneous syndrome characterised by neurologic dysfunction in an infant born at or beyond 35 weeks of gestation, with failure to initiate and maintain respiration. The syndrome is manifested by a reduced level of consciousness or seizures and is frequently associated with reduced reflexes and tone (Coetzee 2018). It is one of the commonest causes of neonatal mortality and severe neurological deficit in children and contributes to ever-increasing medico-legal litigation (Bhorat et al. 2023). In low- and middle-income countries, neonatal encephalopathy accounts for approximately 1 million deaths annually (Pauliah et al. 2013). Globally, 287 000 infants with HIE were estimated to have died in 2010 (Lee et al. 2013). The incidence of HIE differs significantly, with the highest burden prevailing in sub-Saharan Africa (14.9 per 1000 live births) and substantially reduced rates (1.5 per 1000 live births) in high-income countries (Ballot et al. 2020). Charlotte Maxeke Johannesburg Academic Hospital, a tertiary referral centre in South Africa, had HIE rates of 2.3–13.3 per 1000 live births in 2016 (Ballot et al. 2020). In 2011, Chris Hani Baragwanath Academic Hospital (CHBAH) reported the incidence to be 8.7–15.2 per 1000 live births (Bruckmann & Velaphi 2015). A study in the Southern Cape Peninsula reported the incidence to vary from 2.3 to 4.3 per 1000 live births (Horn et al. 2013). Nakwa et al. (2023) reported an incidence of 8.8 per 1000 live births with a mortality of 29%. More than 400 000 infants each year develop neurodevelopmental impairment (NDI) after HIE. In 2010, about 233 000 infants survived with moderate or severe NDI (Lee et al. 2013).

The rate of mortality and the severity of NDI correspond to the severity of HIE, using the Thompson score (Thompson et al. 1997) and Sarnat scoring systems (Sarnat & Sarnat 1976). Neurodevelopmental impairment includes cerebral palsy (CP), cognitive impairment, visual and hearing impairment, epilepsy, attention deficit hyperactivity disorder, autistic spectrum disorders and subtle deficits affecting learning later in childhood (Azzopardi et al. 2014). Infants with mild HIE have been revealed to have few motor or intellectual deficits at preschool age, while those with severe HIE are more heterogeneous in terms of outcomes, having higher mortality and rates of NDI (Van Handel et al. 2007). The risk of severe impairment in at least one domain is high after intrauterine and neonatal insults (Mwaniki et al. 2012).

Therapeutic hypothermia (TH) remains one of the most effective neuroprotective interventions available for HIE (Coetzee 2018). Management with TH has been demonstrated to improve survival and neurodevelopmental outcome at 18 months of age in infants with stage 2 and stage 3 encephalopathy (Mathew, Kaur & Dsouza 2022). This neuroprotective effect has been seen to persist at 6–7 years of age (Coetzee 2018).

Very few studies have examined the developmental profiles of HIE infants. The majority of the studies look at the HIE complications but not at the individual domains affected. Stark, Van der Vyver and Gretschel (2020) managed 30 HIE children from 3 months to 5 years at a secondary hospital in South Africa. Gross motor ability exceeded both cognitive and fine motor skills at 5 years of age. These findings differ with respect to research findings from high-income countries, where fine motor ability supersedes gross motor ability (Mc Guiness et al. 2012). It has been postulated that in less-advantaged communities, children engage in more outdoor play as access to formal schooling and technology is less (Stark et al. 2020). In this study, children with mild HIE developed appropriately, while CP was associated with severe HIE. Those infants with HIE and no major motor disability had a heightened risk of long-term verbal, motor and intellectual deficits at 5 years of age (Stark et al. 2020). In this research, we aimed to describe the range of NDI and the developmental profiles at 1 year of age in infants with stage 2 and 3 HIE, who were treated with and without TH.

### Objectives

This study aimed to describe the neurodevelopmental outcomes of infants with moderate and severe HIE at CHBAH and the proportions with NDI and neurological complications. The study also sought to explore the effect of HIE severity and TH on the neurodevelopmental outcome.

## Research methods and design

### Research approach and design

This was a retrospective, descriptive study of the Neonatal Neurodevelopment Clinic (NNDC) database and records at CHBAH, a tertiary academic hospital in Johannesburg, South Africa.

The primary objective was to document the Griffiths general and subscale quotients (GQ and SQ) and to determine the proportion with NDI, CP, epilepsy, visual and hearing impairment, in 1-year-old children attending the NNDC. The secondary objective was to compare the performance on the Griffith Mental Development Scales (GMDS) in those that were managed with TH and those that were not.

### Setting

Infants with moderate (stage 2) to severe (stage 3) HIE, followed up at the NNDC, between 01 January 2015 and 31 December 2020, were included. Those eligible for the study were those with moderate-to-severe HIE, birth weight ≥ 2000 g, a GMDS assessment at 12 months of age and all CP infants (regardless of GMDS assessment). The NNDC is a weekly clinic that includes neonatologists, neurodevelopmental paediatricians and multidisciplinary rehabilitation professionals (physiotherapists, occupational and speech therapists). The infants are assessed from 6 weeks to 2 years of age. Cranial ultrasounds are repeated at the first visit. Hearing tests are performed at the first visit if not performed in all infants at 12 months and again at 18–24 months of age by GMDS-trained neurodevelopmental paediatricians. At 2 years of age, the children are either discharged or referred to the appropriate specialist clinics for ongoing support.

Clinical examination was performed at every clinic visit with detailed documentation of neurological status, including evidence of epilepsy, CP and visual or hearing impairment. Investigations and specialist referrals were conducted as previously described. CP was further categorised into subtypes (spastic, hypotonic, dyskinetic and mixed) and formally diagnosed at 1 year.

### Neurodevelopmental measures

The GMDS (0–2-year scale) 2nd edition (extended revised) was used to measure neurodevelopmental status. The test has been used routinely at this clinic since 2012, by GMDS-certified clinicians with many years of experience. The test assesses children from birth to 2 years of age, giving an overall GQ score and SQ scores in the five developmental subscales (A–E): Locomotor scale (Scale A), Personal–social scale (Scale B), Hearing and Speech scale (Scale C), Eye and Hand Coordination scale (Scale D) and the Performance scale (Scale E). The GMDS subscales were reported as GQ sub-quotient scores with age equivalents and percentiles.

Neurodevelopmental impairment was defined based on the GQ scores, and the severity of NDI was assessed as mild, moderate or severe. A GQ score of > 85 was classified as appropriate development, 80–84 as mild NDI, 70–79 as moderate NDI and a score less than 70 (<−2 SD) as severe NDI.

The GMDS is used extensively in research settings in South Africa (Amod, Cockcroft and Soellaart 2007; Jacklin & Cockcroft 2013; Luiz, Foxcroft & Stewart 2001). Amod et al. (2007) documented that South African children were similar to their British counterparts with no difference in GQ scores. South African scores from different race and language groups showed good correlation with the British normative values. Positive correlation has been shown between the GMDS (0–2 years) and the Bayley’s Scales of Infant Development-II (BSID-II) (Cirelli, Bickle Graz & Tolsa 2015); the general quotient (GQ) of the GMDS-ER correlates with scores on the Junior South African Individual Scales (JSAIS), as well as academic school performance in South African children (Laughton et al. 2010).

### Population and sample size

**FIGURE 1:**
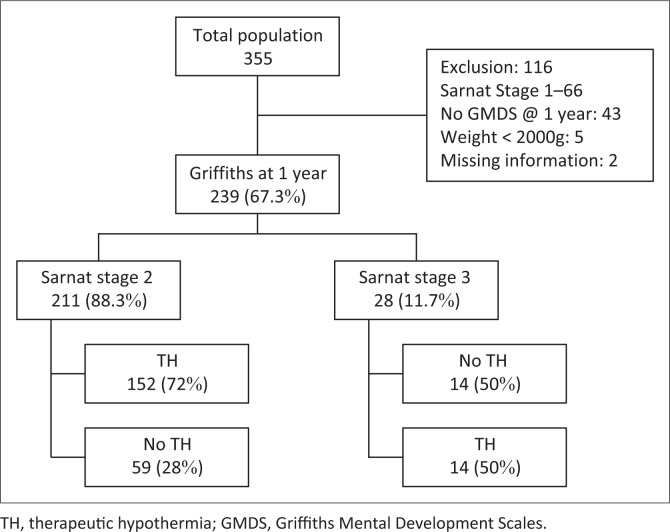
Flow diagram of infants included in the study.

The population comprised all infants seen at NNDC over a 6-year period, between January 2015 and December 2020. There were 355 patients in total, and these included infants with mild, stage 1 HIE (Figure 1). The severity of HIE was assigned with the Sarnat (Appendix 1, Table 1-A1) and Thompson scoring (Appendix 2, Table 1-A2) methods during the neonatal admission. Encephalopathy was graded based on the clinical presentation at birth, including seizures, level of consciousness, autonomic nervous system instability, gross motor system functioning and primitive reflexes. Sarnat stage 2 HIE was defined as moderate and stage 3 HIE as severe. Infants eligible for this study were those with moderate (stage 2) and severe (stage 3) HIE, birth weight ≥ 2000 g, a GMDS assessment at 12 months of age and all CP infants (regardless of GMDS assessment). These criteria resulted in 239 infants – 211 with moderate and 28 with severe HIE (Figure 1). This study aimed to measure the burden of NDI and neurological complications in those with moderate-to-severe HIE.

The CHBAH neonatal unit uses the total body hypothermia (TOBY) criteria for determining the eligibility for TH. The following TH exclusion criteria apply: no spontaneous breathing after 30 min post-resuscitation, heart rate less than 100 b/min at 20 min post-resuscitation, neonates who present beyond 6 h post-delivery and those with major congenital abnormalities. Some infants may not have received TH as a result of these exclusion criteria or the unavailability of TH equipment as a result of demand (Figure 1).

### Data collection and analysis

The demographic data were extracted from neonatal files and the Asphyxia and Neonatal REDCap databases and inserted into an Excel spreadsheet. The GMDS data variables were extracted from the NNDC database.

Data were analysed by using Statistica (version 13). Categorical variables were represented as frequencies and proportions. Continuous variables were expressed as standard deviations and means or interquartile ranges and medians, depending on the data distribution. Comparative statistics were performed to identify factors associated with an abnormal subscale or domain. A chi-square or Fischer exact test was performed to compare categorical variables, and a Mann–Whitney *U* test was performed for comparing skewed continuous variables. A *p*-value of < 0.05 was significant.

### Ethical considerations

Ethical clearance to conduct this study was obtained from the Human Research Ethics Committee of the University of the Witwatersrand (No. M220248). The Asphyxia database registry ethics number is M1511100, and the Neonatal REDCap database number is M151196. Informed consent was waived, as this was a retrospective study. Anonymity was maintained by assigning a study number to the subjects.

## Results

A total of 355 infant files were reviewed. One hundred and sixteen were excluded (details in Figure 1). Two hundred and thirty-nine infants were included; 211 (88.3%) were stage 2, and 28 (11.7%) were stage 3. Therapeutic hypothermia was received by 166 (69.5%) infants, 152 were stage 2 and 14 were stage 3. Therefore, 72% of stage 2 and 50% of stage 3 infants received TH (Figure 1).

The mean gestational age and birth weight at delivery were 38.8 (± 1.7) weeks and 3116 g (± 503.4), respectively. One hundred and thirty-nine infants (58.2%) were male, and more than half of the infants 136 (56.9%) were delivered vaginally. Most infants (86.2%) were born to human immunodeficiency virus (HIV)-negative mothers. There were significantly lower appearance, pulse, grimace, activity, and respiration (APGAR)-scores and higher Thompson scores (TS) in the stage 3 HIE group (Table 1). A greater number of stage 2 HIE patients received TH (*p* = 0.001).

**TABLE 1:** Demographics of infants with moderate-to-severe hypoxic ischaemic encephalopathy.

Variable	All (*n* = 239)					Stage 2 (*n* = 211)					Stage 3 (*n* = 28)					*p*-value
	Mean ± s.d.	*n*	%	Median	IQR	Mean ± s.d.	*n*	%	Median	IQR	Mean ± s.d.	*n*	%	Median	IQR	
Gestational age (weeks)	38.8 ± 1.7	-	-	-	-	38.8 ± 1.8	-	-	-	-	39.2 ± 1.8	-	-	-	-	0.260
Birthweight (g)	3116 ± 503.4	-	-	-	-	3112 ± 485.6	-	-	-	-	3145 ± 631.5	-	-	-	-	0.820
Mode of delivery caesarean section	-	103	43.1	-	-	-	95	45.0	-	-	-	8	28.6	-	-	0.090
HIV unexposed	-	206	86.2	-	-	-	183	86.7	-	-	-	23	82.1	-	-	0.330
Male	-	139	58.2	-	-	-	122	57.8	-	-	-	17	60.7	-	-	0.460
APGAR at 1 min	-	-	-	3	2–5	-	-	-	3	2–5	-	-	-	2	1–4	0.001
APGAR at 5 min	-	-	-	6	5–7	-	-	-	6	5–7	-	-	-	4	3–5	< 0.001
Thompson score (admission)	10 ± 3.2	-	-	-	-	10 ± 3.0	-	-	-	-	14.0 ± 3.7	-	-	-	-	< 0.001
Therapeutic hypothermia	-	166	69.5	-	-	-	152	72.0	-	-	-	14	50.0	-	-	0.010
Age at Griffiths (months)	12.5 ± 1.6	-	-	-	-	12.5 ± 1.6	-	-	-	-	12.2 ± 0.8	-	-	-	-	0.400

s.d., standard deviation; APGAR, appearance, pulse, grimace, activity, and respiration; HIV, human immunodeficiency virus.

**FIGURE 2:**
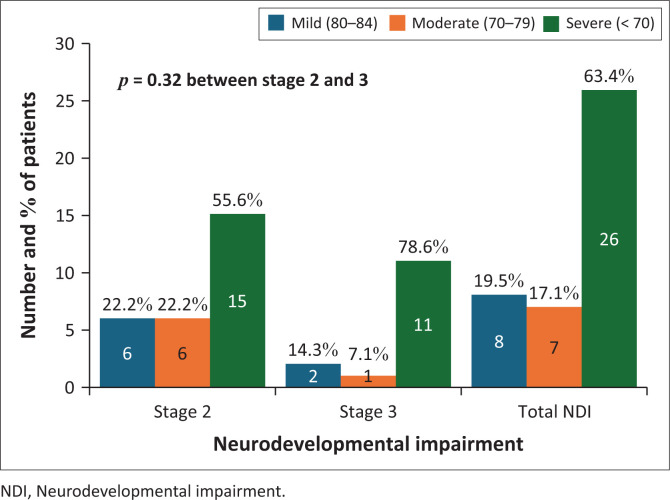
Severity of neurodevelopmental impairment in infants with Sarnat stage 2 and stage 3.

Neurodevelopmental impairment was observed in 17.1% (41) of the whole cohort, of which the majority (63.4%) had severe NDI (GQ < 70), while 17.1% had moderate NDI (GQ 70–79). NDI occurred in 12.7% (27) of the stage 2 (22.2% mild, 22.2% moderate and 55.6% severe NDI) and in 50% (14) of the stage 3 HIE group (14.3% mild, 7.1% moderate and 78.6% severe) as shown in Figure 2. The severity of NDI was not significantly different between the TH and non-TH groups (*p* = 0.25).

Just under half (*n* = 20; 48.8%) of those with NDI had not received TH (Table 2). Sixteen stage 2 NDI infants and five stage 3 received TH. Infants with stage 3 had higher rates of NDI than those with stage 2 (14[50%] vs 27 [12.8%]; *p* < 0.001). Infants with stage 3 who received TH had fewer NDI (5 [35.7%] vs 9 [64.3%]; *p* = 0.005). There were no significant differences in GQ and Griffith subscales between Sarnat stages 2 and 3 in those with NDI, as shown in Table 2. NDI and CP were less frequent in the group that received TH compared to the one that did not (NDI: 21 [12.1%] vs 20 [27.4%]; *p* = 0.005 and CP: 9 [5.4%] vs 13 [17.8%]; *p* = 0.002).

**TABLE 2:** Comparisons of therapeutic hypothermia, general quotient and subscales in Sarnat stages 2 and 3 infants with neurodevelopmental impairment.

Variable	NDI												
	All (stages 2 & 3)				Sarnat stage 2				Sarnat stage 3				*p*-value
	*n* = 41		*n* = 36		*n* = 27		*n* = 26		*n* = 14		*n* = 10		
	*n*	%	Median	IQR	*n*	%	Median	IQR	*n*	%	Median	IQR
TH	21	51	-	-	16	59	-	-	5	36	-	-	0.19
No TH	20	49	-	-	11	41	-	-	9	64	-	-	-
Subscale A (Locomotor)	-	-	52.5	31–71	-	-	55.0	23–73	-	-	52.0	46–63	0.90
Subscale B (Personal-Social)	-	-	60.0	38–74.5	-	-	61.0	38–73	-	-	54.0	37.5–77	0.71
Subscale C (Hearing and language)	-	-	83.0	49–92.5	-	-	85.0	50–93	-	-	72.0	48–92	0.76
Subscale D (Eye-Hand Coordination)	-	-	56.0	31.5–73	-	-	60.0	32–72	-	-	51.5	31–74	0.68
Subscale E (Performance)	-	-	58.5	31–78.5	-	-	61.5	33–73	-	-	50.0	29–83	0.66

TH, therapeutic hypothermia; NDI, neurodevelopmental impairment.

Twenty-two (9.2%) of the infants had CP: 16 (72.7%) Sarnat stage 2 and 6 (27.3%) stage 3. Eight (50%) with stage 2 received TH, and only one (16.7%) infant with stage 3 received TH. More than a third (36.3%) of infants with CP had seizures, six (27.3%) were blind, and three (18.7%) had a hearing impairment. Out of the 9 CP patients that received TH, two (22.2%) were blind, one (11.1%) had hearing impairment, and four (44.4%) had epilepsy.

There was a significantly higher percentage of infants with visual impairment, epilepsy and CP in the stage 3 group versus stage 2 HIE as shown in Table 3. More than a fifth (21.4%) of stage 3 HIE infants were diagnosed with CP; 21.4% had visual impairment, and a quarter (25%) had epilepsy. Stage 2 infants had fewer complications, with 7.5% diagnosed with CP, 0.9% with visual impairment and 6.6% with epilepsy.

**TABLE 3:** Complications in infants with moderate-to-severe hypoxic ischaemic encephalopathy.

Variable	All (*n* = 239)		Stage 2 (*n* = 211)		Stage 3 (*n* = 28)		*p*-value
	*n*	%	*n*	%	*n*	%	
Blind	8	3.3	2	0.9	6	21.4	< 0.001
Hearing impairment	6	3.1	4	1.9	2	7.1	0.190
Epilepsy	21	8.8	14	6.6	7	25.0	0.005
Cerebral palsy	22	9.2	16	7.5	6	21.4	0.020

Note: Type of Cerebral Palsy (CP): Mixed CP-7, Spastic CP-4, Dystonic CP-2, Right hemiplegia-2, and Unspecified-7.

Overall median GQ scores were within normal range for Sarnat stage 2 and 3 infants. However, stage 3 GQ scores (89.5 [69–105]) were significantly lower than stage 2 (GQ 103 [95–109], *p* = 0.002). Stage 2 infants outperformed stage 3 infants in all domains. This difference was significant in all subscales, except in the language domain (subscale C) in Table 4. Therapeutic hypothermia resulted in significantly higher GQ (*p* = 0.002) and subscale scores in Sarnat stages 2 and 3 (data not shown). This effect was also clearly shown in the stage 3 group. Those that did not receive TH had median GQ and subscale scores in the severe NDI range (GQ 69 [43–100] vs 101 [83–107]; *p* = 0.02) as shown in Table 5.

**TABLE 4:** Griffiths subscale for Sarnat stage 2 and Sarnat stage 3.

Variable	All (*n* = 232)		Sarnat stage 2 (*n* = 207)		Sarnat stage 3 (*n* = 25)		*p*-value
	Median	IQR	Median	IQR	Median	IQR	
Age at Griffiths (months)	12	12–13	12	12–13	12.2	12–12.5	0.400
GQ	103	93–109	103	95–109	89.5	69–105	0.002
Subscale A (Locomotor)	101	89–109	101	91–109	96.0	55–104	0.030
Subscale B (Personal-Social)	99	87–105	100	89–105	91.0	60.5–103	0.040
Subscale C (Hearing and Language)	107	100–114	107	100–114	107.0	84–114	0.480
Subscale D (Eye-Hand Coordination)	100	89–111	104	90–111	97.0	56–106	0.030
Subscale E (Performance)	101	90–109	101	92–110	98.0	61.5–104	0.040

GQ, general quotient; IQR, interquartile range.

**TABLE 5:** Griffiths subscale for Sarnat stage 3 for therapeutic hypothermia compared to non-therapeutic hypothermia infants.

Variable	TH (*n* = 14)		Non-TH (*n* = 14)		*p*-value
	Median	IQR	Median	IQR	
Age at Griffiths (months)	12.0	12-13	12.5	12-13	0.25
GQ	101	83-107	69	43-100	0.02
Subscale A	101	77-109	63	46-97	0.02
Subscale B	97	89-105	55	37.5-99	0.03
Subscale C	111	103-115	78	48-110	0.03
Subscale D	100	95-106	55	31-106	0.15
Subscale E	101	84-106	68	29-101	0.04

TH, therapeutic hypothermia; GQ, general quotient; s.d., standard deviation; IQR, interquatile ranges.

## Discussion

This study revealed that infants with stage 2 and stage 3 HIE followed up at CHBAH NNDC over a 6-year period had NDI in 17.1% and CP in 9.2% at 1 year of age. Neurodevelopmental impairment was present in half of the stage 3 and in 12% of stage 2 HIE infants. CP was diagnosed in 21.4% of stage 3 infants and in 7.6% of stage 2 infants. Epilepsy, visual impairment, lower APGAR scores and higher TS were also more frequent in the stage 3 group. This finding is consistent with a vast amount of research globally, which demonstrates that the incidence of long-term disability is related to the severity of HIE.

A Cochrane review in 2007 reported that in infants who survive severe HIE, as many as 80% of them develop significant complications, about 10% – 20% have moderate disabilities, and about 10% of them are healthy. In infants who survive moderate HIE, 30% – 50% of them possibly have significant lasting consequences, and 10% – 20% have slight neurologic disabilities (Evans, Levene & Tsakmakis 2007).

Reported rates of CP after HIE differ but are typically around 10% – 13% among survivors of moderate-to-severe encephalopathy in high-income countries, with dyskinetic CP and spastic quadriplegia being the most common subtypes. Rates of hearing loss are reported to be as high as 17.1% in those with other persistent neurological deficits. Up to 41% of infants diagnosed with HIE have some form of visual impairment in the first year of life (Ahearne et al. 2016).

Mbatha et al. (2021) reported a 32% impairment in 2-year-old infants who received TH at CHBAH, and 6% showed an impairment at 1 year of age. Studies have reported NDI rates between 34.3% and 36% in infants with stage 2 and 3 HIE (Carli, Reiger & Evans 2004; Kachhwaha et al. 2023). In Kathmandu, 45% of the neonatal encephalopathy patients died in the neonatal period, 20% had NDI in survivors at 1 year, and a physical disability rate of 25% was predicted in those with moderate neonatal encephalopathy (Ellis et al. 1999). In Bangladesh, 21.6% of children were found to have normal development and mild disability, all were either stage 1 or stage 2 HIE. Moderate and severe disability were observed in 33% and 25% of the children, respectively (Thayyil et al. 2021). In this study, more infants with stage 3 had NDI than those with stage 2. This finding is similar to that of a report in 2013 at CHBAH, by Sukha, in which infants with stage 3 (100%) had substantially more developmental impairment (*p* = 0.01) than those with stage 2 HIE (52%). More infants with stage 3 had both gross and fine motor delays (Sukka 2013).

The results of this research support the use of TH as a neuroprotective strategy in both stage 2 and stage 3 HIE. Therapeutic hypothermia was associated with fewer NDI (*p* = 0.005) and CP (*p* = 0.002) in these infants. General quotients and SQ median scores were significantly higher in those who received TH and particularly in the stage 3 group.

Many other studies have reported on the benefit of TH in reducing NDI. A systematic review reported that TH decreases developmental impairment and CP in infants with stage 2 and stage 3 (Mathew et al. 2022). A randomised clinical trial by Laptook et al. (2017) showed a 76% probability of any decline in death or disability in the TH compared to the non-TH group. Azzopardi et al. (2014) found that infants with HIE who received TH had markedly reduced risk of CP (21% vs 37%; *p* = 0.03) and that the frequency of moderate-to-severe disability was lower in the TH group (22% vs 37%; *p* = 0.03). Edwards et al. (2010) found that TH improved survival with normal neurological function and decreased the rates of severe neurological disability in survivors. Kachhwaha et al. (2023) found that 85.7% of the infants in the TH group were neurodevelopmentally normal. In the non-TH group, 15 (42.8%) were normal, and 20 (57.2%) had some neurodevelopmental abnormality. Gano et al. (2014) reported that the proportion of patients with NDI at 12 months was significantly lower (*p* < 0.05) in the TH group (9.43%) than in the non-TH group (36%). Ellis et al. (1999) found that the majority with NDI had severe NDI, and 78% of those children had CP. In this study, infants who received TH were less likely to develop NDI and CP. The rates of CP were lower as the moribund infants were excluded from receiving TH, and the more severe infants died in the neonatal period.

General quotients and sub-quotients were in the normal range for the group as a whole at 1 year. All group comparisons revealed differences in all subscales, except for subscale C. Language testing at 12 months may be less sensitive than later testing, as the first year milestones mostly relate to prelinguistic skills and communicative intent. The complexity of language, both vocabulary and receptive language, increases rapidly during the second year. Vocabulary development presents a lexical spurt at around 17 months (Serrat-Sellabona et al. 2021). Language delays, especially subtle deficits, are more likely to be detected later when standardised testing is performed. Items recorded on the GMDS at 12 months include understanding 1 to 2 single words, singing, and observing the response to name and inhibitory words, and some rely on the maternal report. Mbatha et al. reported higher rates of NDI on GMDS testing at 18–24 months than at 12 months. Therefore, standardised testing at 2 years and beyond provides a deeper understanding of subtle deficits. Language is therefore unlikely to be a sensitive indicator of neurodevelopmental delay at 1 year of age using the GMDS. Specific standardised language tools should be considered for testing at 12 months in future studies.

A third of stage 2 and stage 3 infants who received TH in the same institution had moderate-to-severe NDI at 18–24 months. A greater number survived with mild or no impairment at 2 years (Mbatha et al. 2021). These findings highlight the significance of continuous developmental assessment beyond the first year of life. Normal scores on the GMDS at 1 year or 2 years do not always predict a good outcome at 6 years and 7 years. An abnormal score is most likely to be associated with substandard academic performance at school age (Barnett et al. 2004). Early neurodevelopmental assessment in the first 2 years of life does not rule out neurological, cognitive or perceptual motor abnormalities at school-going age. The sensitivity values for the GMDS were marginally higher at 2 years than at 1 year (90% vs 76%) (Barnett et al. 2004). An understanding of the outcome of this category of children is essential because these subtle deficits can affect academic achievement and cause behavioural problems in the future. Failure to identify such children in infancy results in a missed opportunity for early intervention, which may help them cope better at home and at school (Barnett et al. 2004).

The strength of this study is that it investigated data which was collected over a 6-year period at the largest hospital in sub-Saharan Africa. The sample was large, and standardised GMDS testing was routinely performed in all patients, resulting in high-quality data. The limitations of this study were that this was a retrospective descriptive study and, therefore, could not evaluate causation, but only associations. The severe HIE group had far smaller numbers than the moderate group had; 50% had NDI, and 21.4% had CP. These results, although encouraging when compared to other studies, which report rates of NDI around 80% for severe HIE, should be interpreted cautiously. These infants represent a small sample in that the most severe HIE had died. They also had lower rates of TH in this group as compared to those in the moderate HIE sample. The infant GMDS at 12 months may not detect subtle language deficits and therefore, analysis at later ages or alternative standardised tools should be considered.

## Conclusion

Neurodevelopmental impairment and CP were observed in 17.1% (41) and 9.2% (22) of the cohort, which compares favourably with the rates in other countries. The GQ and SQ scores were in the average range for the cohort with equivalent profiles across the five domains. Therapeutic hypothermia and moderate HIE were associated with decreased CP and NDI. Neurodevelopmental assessments at 2 years and beyond are necessary to determine longer-term outcomes and subtle deficits.

## Appendix 1

**Table 1-A1:** Sarnat classification.

Variables	Stage I (Mild)	Stage II (Moderate)	Stage III (Severe)
Consciousness	Hyperalert	Lethargic or obtunded	Stupor or coma
Activity	Normal	Decreased	Absent
**Neuromuscular control**			
Muscle tone	Normal	Mild hypotonia	Flaccid
Posture	Mild distal flexion	Strong distal flexion	Intermittent decerebration
Stretch reflexes	Overactive	Overactive	Decreased or absent
**Primitive reflexes**			
Sucking	Weak	Weak or absent	Absent
Moro	Strong	Weak incomplete/strong	Absent
Tonic neck	Slight		Absent
**Autonomic function**			
Pupils	Dilated	Constricted	Variable, unequal
Heart rate	Tachycardia	Bradycardia	Variable

## Appendix 2

**Table 1-A2:** Thompson score.

Sign	Score			
	0	1	2	3
Tone	Normal	Hyper	Hypo	Flaccid
Level of consciousness	Normal	Hyper alert, stare	Lethargic	Comatose
Fits	None	Infrequent < 3/day	Frequent > 2/day	-
Posture	Normal	Fisting, cycling	Strong, distal flexion	Decerebrate
Moro-reflex	Normal	Partial	Absent	-
Grasp-reflex	Normal	Poor	Absent	-
Suck-reflex	Normal	Poor	Absent	-
Respiratory pattern	Normal	Hyperventilation	Brief apnoea	IPPV (apnoea)
Fontanelle	Normal	Full, not tense	Tense	-

IPPV, intermittent positive pressure ventilation.
